# East Texas Medico=Chirurgical Society

**Published:** 1902-05

**Authors:** 


### East Texas Medico-Chirurgical Society.

The second annual meeting of the East Texas Medico-Chirurgical Society will be held Thursday and Friday, May 29 and 30, 1902, at Palestine, Texas.

Officers: Dr. W. C. Lipscomb, President, Crockett, Texas; Dr. A. L. Hathcock, First Vice-President, Palestine, Texas; Dr. E. P. Murdock, Second Vice-President, Oakwoods, Texas; Dr. J. M. Colly, Treasurer, Palestine, Texas; Dr. E. E. Guinn, Secretary, Rusk, Texas.

Program: [We regret that for want of space we are compelled to omit the program, which is very full and will be most interesting.]

It will be seen that this progressive society has a strong Committee on Enforcement of New Medical Law: Dr. J. N. Gee, Bethel, Anderson county; Dr. E. E. Bryson, Pittsburg, Camp county; Dr. J. M. Crawford, Alto, Cherokee county; Dr. T. J. Bell, Tyler, Smith county; Dr. R. C. Bristow, Athens, Henderson county; Dr. S. S. Barnett, Kilgore, Gregg county; Dr. E. P. Murdock, Oakwoods, Leon county; Dr. J. L. Hall, Crockett, Houston county; Dr. W. I. M. Smith, Nocogdoches, Nacogdoches county.

Doctor, if you are not a member of this body, send the secretary $1.00, together with information concerning your graduation, and become one.

